# Lysosomal size matters

**DOI:** 10.1111/tra.12714

**Published:** 2019-12-06

**Authors:** Mariana E. G. de Araujo, Gudrun Liebscher, Michael W. Hess, Lukas A. Huber

**Affiliations:** ^1^ Institute of Cell Biology, Biocenter Medical University of Innsbruck Innsbruck Austria; ^2^ Institute of Histology and Embryology Medical University of Innsbruck Innsbruck Austria; ^3^ Austrian Drug Screening Institute, ADSI Innsbruck Austria

**Keywords:** AMPK, autolysosome, autophagy, BORC, endolysosome, fission, fusion, LAMTOR, late endosome, lysosomal acidification, lysosomal reformation, lysosomal storage disorders, lysosome, PtdIns(4,5)P_2_, PIKfyve, PtdIns(3)P, PtdIns(3,5)P_2_, V‐ATPase

## Abstract

Lysosomes are key cellular catabolic centers that also perform fundamental metabolic, signaling and quality control functions. Lysosomes are not static and they respond dynamically to intra‐ and extracellular stimuli triggering changes in organelle numbers, size and position. Such physical changes have a strong impact on lysosomal activity ultimately influencing cellular homeostasis. In this review, we summarize the current knowledge on lysosomal size regulation, on its physiological role(s) and association to several disease conditions.

## Graphical abstract

Over the last decade, we witnessed an unprecedented interest in lysosomes, most prominently as catabolic centers and as intracellular signaling platforms. Interestingly, lysosomal activity is strongly dependent on organelle numbers, size and position. The dynamic nature of lysosomes has been extensively documented but the importance of lysosomal size is only now coming to light. In this review, we summarize the current knowledge on lysosomal size regulation, its physiological importance and association with disease conditions.

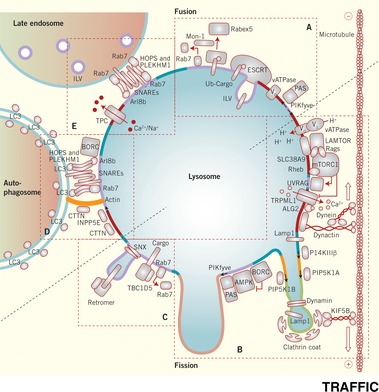

## 1 INTRODUCTION

Initially described by Christian de Duve,^1^, ^2^ lysosomes are key mediators of protein degradation with a pivotal role in the coordination of cellular metabolism and intracellular signaling.^3^ Lysosomes participate in many biological processes, including antigen presentation, plasma membrane repair, exosome release, cellular adhesion and migration, apoptosis, gene regulation, tumor invasion and metastasis (reviewed in References 3, 4, 5).

The complexity of lysosomes is emphasized by data from proteome analyses: ion channels and transporters regulate the luminal ion composition,^6^, ^7^ a dedicated H+‐ATPase maintains the luminal acidic pH,^8^, ^9^ tethering factors and SNARE proteins at the membrane control fission and fusion with communicating cellular compartments.^10^, ^11^ In addition, around 200 glycosylated and non‐glycosylated integral membranes proteins fulfill several critical functions including, for example, the transport of metabolites and the protection of the organelle membrane from degradation.^5^, ^12^

In response to diverse intra‐ and extracellular stimuli, lysosomes continuously adjust their numbers, size and position. It has been previously demonstrated that the localization and motility of those organelles significantly affect their activity.^13^, ^14^ Compromised lysosomal positioning is associated with detrimental effects present in several disease conditions, most prominently cancer.^15^, ^16^, ^17^ It is also intuitively clear that changes in lysosome number influence the degree to which a certain function can be performed. Interestingly, among the different parameters, the contribution of lysosomal size remained somewhat obscure. Does size matter and if so, to what extent is it linked to organelle numbers?

Various terms are used to differentiate specific intracellular compartments, including organelles of the endocytic and autophagic routes. For clarity reasons, we would like to define the terms used in this review. Late endosomes fuse with terminal lysosomes forming a hybrid compartment called endolysosome.^18^ Endolysosomes and terminal lysosomes coexist in a dynamic equilibrium that is maintained by lysosomal reformation.^19^ Unless otherwise stated, we use the term lysosome to identify both endolysosomes and lysosomes, jointly corresponding to very late stages—frequently the very end of the endocytic pathway. Whenever necessary, endolysosomes and terminal lysosomes are discriminated as such. As far as autophagy is concerned, autolysosomes arise from the fusion of autophagosomes with lysosomes.^20^

**Figure 1:**
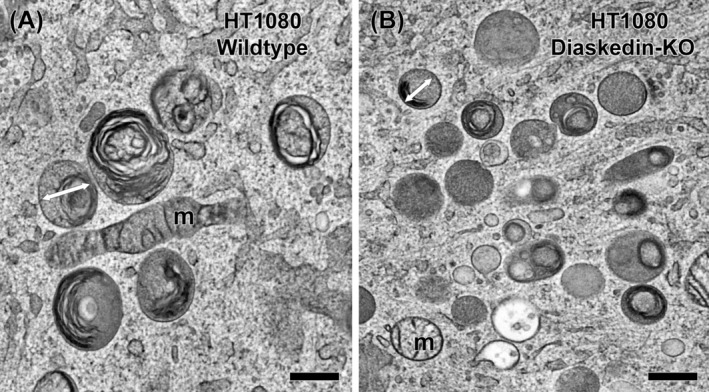
Electron micrographs of snap‐frozen lysosomes displaying general morphology and size variations of the almost spherical organelles in HT1080 human fibrosarcoma cells. The term lysosome is used in a broad sense comprising both terminal lysosomes resulting from cargo endocytosis as well as autolysosomes. Those organelles are characterized by an opaque matrix, containing frequently more or less degraded (membrane) material and/or a completely electron‐dense core. A, Under wildtype conditions the lysosomal diameter (white arrow) is on average 400 nm. B, After deletion of BLOC‐1 related complex (BORC) the mean diameter decreases to approximately 300 nm; m = mitochondrion; scale bar = 400 nm

In the following, we recapitulate general aspects of lysosomal geometry and size, based on two‐dimensional (2D) electron microscopy (EM) snapshots, morphometric measurements, counts and calculations. As already demonstrated by the pioneers in the field,^21^ terminal lysosomes are typically spherical organelles, appearing as round or ovoid/elliptical section profiles (eg, Figure 1A), with occasional, short‐lived tubular extensions (eg, during lysosome reformation^22^). For practical reasons, we exclude the less common, tube‐shaped lysosomes^22^, ^23^ from the following considerations. EM‐based morphometry^24^ is unique in providing a solid basis for the precise detection of even moderate qualitative and quantitative alterations of organelle ultrastructure, especially when cryo‐fixation methods^25^ are used for sample preparation. Cryo‐fixation also traps transient and/or labile membrane configurations^26^, ^27^ and efficiently prevents uneven artifactual organelle shrinkage, that selectively affects different maturation stages of endocytic compartments.^28^ To mention an exemplary case, HT1080 cells (human fibrosarcoma cell line carrying an N‐ras mutation^29^) have globular lysosomes with an average diameter of 410 nm.^30^ In contrast, lysosomes of HT1080 cells, where components of the BORC (BLOC‐1 related complex) complex have been deleted by CRISPR/Cas9 genome editing, are only 311 nm wide (Figure 1 and Reference 30). Further discussion of this difference follows later in this article. Although a ≈25%‐reduction of organelle diameter might appear minor at first glance, simple arithmetic allows the conversion of the 2D‐value into 3D‐reality. As illustrated in Table 1, a 25% diameter reduction yields about half the surface area or the volume of spherical organelles—a quite relevant physiological difference. Bearing this basic geometry in mind, we will now discuss additional aspects of lysosomal size variations.

**Table 1:** Estimation of lysosomal (LY) size changes as calculated from ultrastructure morphometry measurements of cryo‐fixed samples

	Model calculation for spherical organelles	
	Full size = 100%	Reduced size
Diameter	1.000	0.750 = 75%
Surface area	3.142	1.767 = 56%
Volume	0.524	0.221 = 42%

	Biological Example 1: LY in HT1080	
	HT1080 WT	HT1080 Diaskedin KO
Diameter (nm)	410 nm	311 nm = 76%
Surface area (μm^2^)	0.528 μm^2^	0.302 μm^2^ = 58%
Volume (μm^3^)	0.036 μm^3^	0.016 μm^3^ = 44%

	Biological Example 2: LY in HeLa	
	HeLa WT	HeLa Diaskedin KO
Diameter (nm)	339 nm	258 nm = 76%
Surface Area (μm^2^)	0.363 μm^2^	0.212 μm^2^ = 58%
Volume (μm^3^)	0.021 μm^3^	0.009 μm^3^ = 43%

*Note*: Mean values from Reference^30^; LY are assumed here as spheres.

First, size represents a physical constraint determining the ratio between membrane surface area and organelle volume. Early work on the yeast syntaxin homolog Pep12 indicated that this SNARE is essential for fusion of endocytic vesicles. Pep12 deletion is phenotypically characterized by an accumulation of small, 40 to 50 nm vesicles in the cytoplasm and an inflation of the vacuole,^31^ the yeast equivalent of lysosomes and plant vacuoles. Importantly, the limited volume of the endosomes effectively prevented intraluminal vesicle formation,^32^ blocking transport of ubiquitinated cargo towards the vacuole.^31^, ^32^ In addition, this structural defect also impaired the recruitment of hydrolases, leading to an acidification defect and concomitant enlargement of the vacuole.

Second, size regulates dynamics. In 2014, Bandyopadhyay et al addressed the influence of lysosome diameter on the transport of organelles in living cells.^33^ In brief, active transport occurred within a broad range of velocities and was not affected by organelle size. In contrast, the diffusive component of lysosomal movement inversely correlated with the organelle size.^33^ In addition, it has been shown that increased lysosomal size negatively influences exocytosis.^34^ Enlarged lysosomes in fibroblasts from Chediak‐Higashi patients as well as from beige mice displayed reduced exocytosis that could be reverted by treatment with E‐64d, a protease inhibitor, reducing lysosomal size in those cells.^34^ Mechanistically, E‐64d treatment protects PKC from ceramide‐induced, calpain‐mediated degradation thereby reducing lysosomal size.^35^ The protease inhibitor also increases lysosomal elastase and cathepsin G activity.^35^ These results imply that the impaired exocytosis observed could be attributed to the abnormal size of lysosomes.

**Figure 2:**
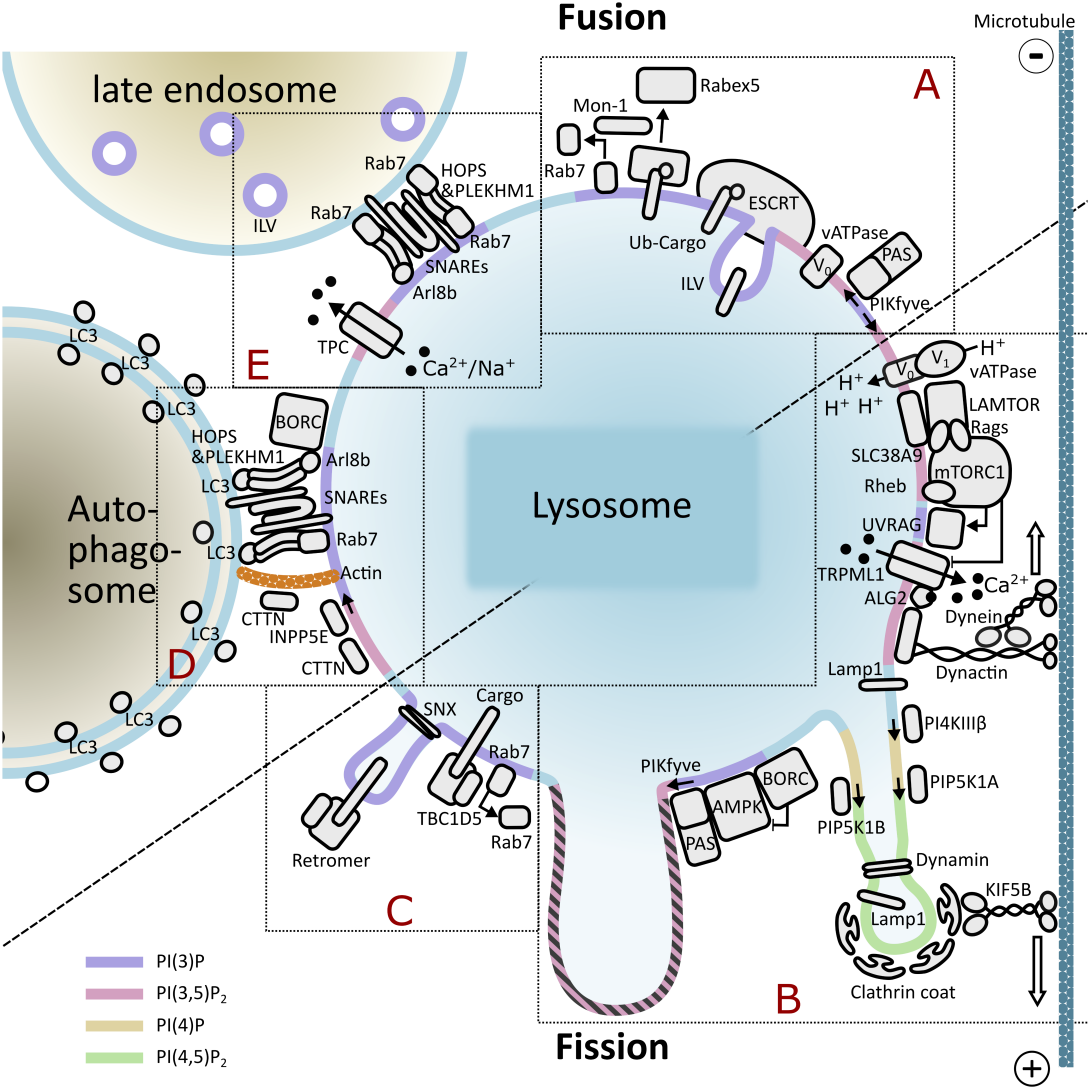
Comprehensive scheme of key components and protein machineries of late endosomes/lysosomes/autophagosomes and their involvement in the regulation of organelle size. For the purpose of graphical simplification in this figure, the term lysosome refers to endolysosomes, terminal lysosomes and autolysosomes. The image has been subdivided into different boxes annotated with red letters, A‐E, so as to link the machineries to the respective text paragraphs. The localization of PIP(3,5)P_2_ to lysosomal reformation tubules, as postulated by References 27, 30 is still hypothetical; this is indicated by the use of a dashed line in pink and gray for this feature

Importantly, since lysosomal size is altered in several human diseases this might directly or indirectly contribute to the pathophysiology of the different conditions. Below, we summarize the current knowledge of factors contributing to the regulation of lysosomal size and frequency and describe human disorders associated with alterations of endolysosome‐, phagolysosome‐ and/or lysosome‐size. In Figure 2 we schematically guide the reader through the following chapters and introduce key components and protein machineries of late endosomes/lysosomes/autophagosomes and their involvement in the regulation of organelle size.

## 2 V‐ATPASE AND ORGANELLE ACIDIFICATION

Vacuolar proton‐translocating ATPases (V‐ATPases) are highly conserved, ATP‐driven proton pumps that—as the name indicates—acidify endocytic, secretory and autophagic organelles. Reversible association of the cytoplasmic, peripheral V_1_ domain with the integral membrane‐bound V_0_ domain regulates their function. The assembly of the V‐ATPase promotes organelle acidification that is fundamental for the controlled dissociation of ligand/cargo and enzyme/cargo pairs and the correct targeting of the components to either degradation or recycling. In specialized cells, V‐ATPases actively contribute to synaptic transmission, activity of secretory granules and antigen presentation.

Importantly, the rate of acidification is not uniform among terminal endocytic organelles, strongly depending on the organelle's position within a cell^36^ and on its role within the reformation cycle.^19^ In brief, terminal lysosomes are only moderately acidic, smaller and acid‐hydrolase inactive, whereas endolysosomes tend to be bigger, acidify and are catabolically active. Although not formally proven, it has been proposed that pH fluctuations might facilitate the function of lysosomal enzymes with neutral or alkaline pH optima.^37^

Inhibition of the V‐ATPase blocks the transport of endocytic markers from early endosomes to more acidic organelles. In other words, the V‐ATPase is required for the formation of intermediate compartments between early and late endosomes, generally designated as multivesicular bodies (MVBs).^38^ In addition, it has been proposed since two decades that the V‐ATPase regulates both fission and fusion events, thereby contributing to the control of the lysosome/vacuole size (Figure 2A, B). Interestingly, while it is well accepted that organelle fission requires the acidifying activity of a fully assembled V‐ATPase,^39^, ^40^, ^41^, ^42^, ^43^ the importance of the V‐ATPase in lysosomal fusion is still a matter of debate. In *Saccharomyces cerevisiae*, the V‐ATPase was shown to fulfill a structural function during membrane fusion. In brief, upon SNARE pairing the V_0_ domains present in adjacent membranes associate in a trans‐complex. Complex formation generates a proteolipid fusion pore that facilitates membrane fusion.^44^, ^45^, ^46^ Although this view has been strengthened by subsequent reports,^43^, ^47^ the mechanistic details of this regulation and the importance of acidification in fusion remain controversial.^44^, ^48^ In higher eukaryotes, the V‐ATPase seems to fulfill a regulatory role, not being intrinsically necessary for all lysosomal fusion events. As an example, it has been demonstrated that the V_0_ domain of the V‐ATPase regulates exocytosis of synaptic vesicles in higher eukaryotes.^49^, ^50^ In contrast, in macrophages the V_0_ domain was not required for phagosome‐lysosome fusion.^51^

Mammary gland involution provides another impressive example of the importance of acidification in the control of lysosomal size and numbers. This regulated tissue remodeling process strongly depends on lysosomal acidification and biogenesis to mediate the conversion of the lactating gland to a prepregnant state. Recent work revealed that loss of the Zinc transport 2 (ZnT2) prevents the assembly of the V‐ATPase on lysosomes, reducing the number of organelles as well as their size and ultimately impairs lysosomal mediated cell death that would be required for involution.^52^, ^53^

## 3 PI METABOLISM AND ORGANELLE SIZE

Weak base compounds like Chloroquine were some of the first agents found to trigger swelling of lysosomes,^54^ followed by experiments using Wortmannin.^55^ In melanoma cells, the latter one, a PI 3‐kinase inhibitor, leads to inflation of the organelles in a process dependent on endocytic membrane influx. Importantly, Wortmannin inhibition also triggers a decrease in the number of intraluminal vesicles of MVBs.^55^ These observations, highlighted the importance of phosphatidylinositol‐3phosphate (PtdIns(3)P) the product of PI3K activity, in maintaining the size and structure of late endocytic organelles. Interestingly, PtdIns(3)P levels also control autophagosomal size.^53^, ^56^

Two decades ago, PtdIns(3)P was found to accumulate within MVBs.^57^ Among the different PtdIns(3)P effectors, the *hepatoc*y*te* growth factor‐regulated tyrosine kinase substrate (HRS) is a member of the endosomal sorting complex required for transport complex 0 (ESCRT‐0), contributing to the sorting of cargo marked with ubiquitin into intraluminal vesicles,^55^, ^58^, ^59^, ^60^ (Figure 2A).

In addition, PtdIns(3)P regulates the recruitment of the Mon1 complex, responsible for the removal of Rabex5 from the endosomal membrane. The Mon1 complex also interacts with the homotypic fusion and protein sorting complex (HOPS) contributing to the timely recruitment of Rab7 and the promotion of early to late endosomal transition.^61^ PI3K and Rab7 cooperate in a coordinated effort to control retromer dependent retrograde sorting of cargoes from the late endosome to the trans‐Golgi network (TGN) and the plasma membrane, (Figure 2C). In brief, retromer recruitment to the organelle membrane is mediated by a 2‐fold recognition system. On one hand, the complex is recognized by sorting nexins, bona fide PtdIns(3)P effectors, containing a phox homology domain. On the other hand, retromer recruitment also requires the interaction between its VPS35 subunit and RAB7‐GTP.^62^, ^63^ In turn, Rab7 activity is kept in check by TBC1D5, a Rab7 GAP that depends on retromer for its membrane association (Figure 2C ).^64^ Of note, Rab7 hyperactivation, either by overexpression or because of impairment of TBC1D5 function, leads to the formation of enlarged endolysosomes, increased transport of cargo along retromer dependent routes and a decrease of mitophagy.^65^, ^66^ Mechanistically, the increase in Rab7 prevents autophagic lysosome reformation (ALR), thereby triggering the accumulation of enlarged organelles.^22^

Like HRS, PIKfyve (also known as *1‐phosphatidylinositol 3‐phosphate 5‐kinase*) is a PtdIns(3)P effector that contains an FYVE domain critical for its membrane localization.^67^ PIKfyve is the sole enzyme known so far capable of catalyzing phosphatidylinositol3,5‐bisphosphate (PtdIns(3,5)P_2_) out of its precursor. PIKfyve activity triggers a local depletion of PtdIns(3)P and PtdIns(3)P‐dependent proteins and the recruitment of PtdIns(3,5)P_2_ effectors to the organelle membrane, (Figure 2A,B).

Earlier studies in *Saccharomyces cerevisiae* have identified the importance of PtdIns(3,5)P_2_ in membrane trafficking.^39^, ^40^, ^68^ In brief, deletion of yeast *fab1*, the homolog of human PIKfyve, triggered a severe growth defect and was characterized by the presence of enlarged vacuoles that occupied the majority of the cell. The yeast data led to a model in which PtdIns(3,5)P_2_ levels are carefully tuned to maintain the turnover of vacuolar membranes to less mature endocytic compartments, hence, contributing towards the regulation of the organelle's size.^68^ Of note, the fact that *fab1* mutants display reduced numbers of intraluminal vesicles and Vps24 (a subunit of ESCRT‐III) interacts with PtdIns(3,5)P_2_, emphasizes again a tight interconnection in the activities mediated by different PtdIns(3)P effectors,^69^ (Figure 2A).

PIKfyve does not act alone. This phosphoinositide kinase associates with a preassembled arPIKfyve (Vac14) and sac3 (Fig4) complex. The ternary complex is known as PAS (PIKfyve‐ArPIKfyve‐Sac3) complex.^70^ ArPIKfyve, the structural mediator, maintains the integrity of the complex via homo and heteromeric interactions with the remaining subunits.^71^ Overall, the PAS complex is organized to provide optimal PIKfyve functionality.^72^ In addition, PAS contains two enzymes with opposing effects, PIKfyve that catalyzes PtdIns(3,5)P_2_ production, and sac3 that converts it into PtdIns(3)P. A peculiarity of PAS is that the presence of the phosphatase sac3 is also required for PIKfyve activation.^73^, ^74^

PtdIns(3,5)P_2_ is a low abundant lipid, typically accounting for 0.04% to 0.08% of total inositol phospholipids present in human cells.^75^ Despite the minute expression levels, PtdIns(3,5)P_2_ is an essential lipid species and its depletion in mice, *Drosophila melanogaster* and *Caenorhabditis elegans* is embryonic lethal.^76^, ^77^, ^78^, ^79^ In mammalian cells, ablation of PtdIns(3,5)P_2_ induces an imbalance in endosomal membrane homeostasis resulting in the enlargement of both early and late endosomal/lysosomal compartments.^80^ Interestingly, too much PtdIns(3,5)*P*_2_ can also be detrimental to lysosomal homeostasis. In 2016, Hasegawa et al uncovered INPP5E as the enzyme mediating the conversion of PtdIns(3,5)*P*_2_ to PtdIns(3)P. Inhibition of INPP5E triggered the accumulation of PtdIns(3,5)*P*_2_ and compromised autophagosome‐lysosome fusion,^81^, ^82^ (Figure 2D).

Several seven‐bladed β‐propeller proteins (WD40 domain) have been identified as PtdIns(3,5)*P*_2_ interactors, including the yeast proteins Atg18, Cti6, Tup1 and in higher eukaryotes (murine adipocytes) Raptor.^83^, ^84^, ^85^ Of note, deletion of *Atg18* triggers enlargement of the vacuole that is phenotypically indistinguishable from *fab1/*PIKfyve ablation.^84^ In addition, the presence of PtdIns(3,5)*P*_2_ is recognized by the PX domain containing sorting nexins 1 and 2 (SNX1 and SNX2)^86^, ^87^ and the sec14 domain containing clavesin.^88^ Interestingly, clavesin knockdown in neurons also enlarges lysosomal‐associated membrane protein 1 (LAMP1) positive structures.^88^ In plants, PtdIns(3,5)*P*_2_ regulates polarized cell growth, by recruiting class II formins (via PTEN domain), to cortical actin.^89^ The identification of cortactin as a direct binding partner of late endosomal PtdIns(3,5)*P*_2_ in human cells provides a further testimony of the importance of this lipid in the control of actin dynamics. In brief, the interaction between PtdIns(3,5)*P*_2_ and cortactin negatively regulates the association of the latter to nascent organelle bound actin filaments, thereby decreasing the rate of branched actin assembly and promoting local actin turnover^90^ (Figure 2D).

Two independent lines of evidence imply that defective fission might be the common denominator, mechanistically explaining the phenotypes observed upon PIKfyve inhibition or depletion of PtdIns(3,5)P_2_. First, defects in trafficking of retromer‐dependent and ‐independent cargo from endosomes to the TGN have been reported upon PIKfyve inhibition.^80^, ^91^, ^92^, ^93^ Second, PIKfyve inhibition was shown to prevent lysosomal reformation with concomitant increase in enlarged endolysosomes.^27^ Under these circumstances, organelles coalesce, thereby decreasing their numbers and increasing their size.^94^ Both of these processes, retrograde transport and lysosomal reformation utterly, depend on efficient fission. In contrast, the contribution of an imbalanced endocytic pathway to PIKfyve ablation phenotypes seems still a matter of debate with some authors claiming a block in the degradation of both EGFR and c‐Met,^91^ whereas others observe no clear defects.^80^

Mechanistically, the size of an organelle is to a large extent dependent on the rate of membrane exchange, separated in the relative contributions of fusion and fission processes. In lysosomes, high levels of juxta‐organellar calcium, released from the intraluminal pool, were detected in the vicinity of fusion/fission sites. Interestingly, PtdIns(3,5)P_2_ interacts with the mucolipin transient receptor potential channels (TRPMLs), activating them and promoting calcium transport across the organelle membrane^95^ (Figure 2B). In a reverse manner, PtdIns(4,5)P_2_ was shown to inhibit TRPML1 activity.^96^ Of note, TRPML1 deficient cells exhibit enlarged lysosomes/vacuoles and trafficking defects reminiscent of those observed by depletion of PtdIns(3,5)P_2_ and overexpression of TRPML1 were sufficient to revert PtdIns(3,5)P_2_ deficiency phenotype.^95^ This regulatory pathway is conserved in *Saccharomyces cerevisiae*, where PIKfyve regulates vacuolar size through TRPML1.^97^ In 2016, Li et al extended this epistatic alignment and revealed that the PtdIns(3,5)P_2_‐TRPML1‐ALG‐2‐dynein signaling cascade is required for lysosome tubulation and reformation^98^ (Figure 2B). Interestingly, mTORC1 phosphorylation of TRPML1 prevents the activation of the calcium channel when nutrients are abundant, thereby restricting the action of TRPML1 to starvation conditions.^99^, ^100^ In turn, TRPML1 and calmodulin activities are required for mTORC1 reactivation during prolonged starvation^100^, ^101^ (Figure 2B ). Finally, TRPML1 levels are compromised in patients suffering from Mucolipidosis type IV, a rare lysosomal storage disease triggered by mutations in the mucolipin 1 (*TRPML1*) gene. For more details on mucolipidosis type IV please see below.

TPC are lysosomal two‐pore channel proteins that were initially identified as mediators of nicotinic acid adenine dinucleotide phosphate (NAADP)‐dependent calcium release.^7^ Later, TPCs were shown to transport sodium upon activation by PtdIns(3,5)P_2_.^102^, ^103^ The ion selectivity properties of TPC remains a controversial topic and might be dependent on the experimental conditions.^104^ Importantly, TPC‐mediated calcium transport was shown to rapidly reduce and reverse the membrane potential, thereby promoting organelle fusion (Figure 2E ). In accordance to its mode of action, TPC overexpression induced enlarged lysosomes.^102^ Surprisingly, unlike TRPMLs, the expression pattern of TPC is restricted to certain cell types indicating that the relative contribution of different ions and transporters to the regulation of lysosomal membrane characteristics might be cell type dependent.

In *Saccharomyces cerevisiae*, genetically ablated *fab1* (PIKfyve), vac14 (arPIKfyve) and *vac7* cells have vacuoles with neutral pH, a defect that appears to occur independently of vacuole enlargement^76^, ^77^ and of a possible mislocalization of the V‐ATPase.^105^ A similar phenomenon was observed in lysosomes of *Caenorhabditis elegans* and *Drosophila melanogaster* cells encoding a partially functional PtdIns(3)*P* 5‐kinase.^40^ Apparently, at least in yeast, the levels of PtdIns(3,5)*P*_2_ required to support acidification are lower than those required to maintain organelle morphology, indicating that under certain conditions, vacuoles may increase in size without an accompanying acidification defect.^106^ In other words, PtdIns(3,5)*P*_2_ controls organelle characteristics by different mechanisms, one of them being acidification. Mechanistically, PtdIns(3,5)*P*_2_ binds directly the V_0_ integral membrane domain of the V‐ATPase and at least in yeast, increased PtdIns(3,5)*P*_2_, driven as a response to salt stress, recruits the N‐terminal region of V_0_ subunit Vph1p from the cytosol to organelle membranes.^107^ This event favors V_1_‐V_0_ assembly and thus increases V‐ATPase activity.^107^

## 4 AUTOPHAGIC LYSOSOMAL REFORMATION

Autophagy is a catabolic process activated under starvation conditions by which dispensable cytoplasmic proteins and organelles are degraded to recycle amino acids required to maintain cell survival. From a mechanistic perspective, starvation triggers mTORC1 inactivation and the initiation of the autophagy program. In addition, starvation activates the general amino acid control (GAAC) pathway, upregulating amino acid transporters at the plasma membrane.^108^ The increased uptake of amino acids through GAAC, together with the release of free amino acids from autolysosomes upon autophagy induction, are responsible for the temporary reactivation of mTORC1 under prolonged starvation conditions. Increased mTORC1 activity is fundamental to initiate ALR, necessary to replenish the number of catabolically active lysosomes.^22^, ^42^ Mechanistically, this process is mediated by a complex formed by the ultraviolet (UV) radiation resistance‐associated gene protein (UVRAG) and VPS34, that stimulates the production of PtdIns(3)P on lysosomes^109^ (Figure 2B ).

A decade ago, scientists described for the first‐time tubular structures emanating from autolysosomes with vesicles budding from the tubule's tip and thereby established ALR (Figure 2B). The tubular and vesicular structures were positive for the bona fide lysosomal marker LAMP1, lacked the autophagosomal marker LC3, and contained no degrading cargo. Interestingly, emerging vesicles (now called proto‐lysosomes) progressively acquired a more acidic pH and increased their degradative capacity, in other words, they matured into fully functional lysosomes.^22^

Meanwhile, the minimal requirements for the execution of ALR have been identified: PIP5K1B catalyzes the conversion of PtdIns(4)P to PtdIns(4,5)P_2_, which is the central molecule for ALR. Clathrin and PtdIns(4,5)P_2_, and their enrichment on autolysosomal membrane domains, lead to the recruitment of the motor protein KIF5B. In turn, KIF5B actively pulls on the membrane, driving the tubulation process^110^, ^111^ (Figure 2B). KIF5B serves different functions in lysosomes and autolysosomes—also here, size matters. Smaller vesicles (100‐200 nm) can be transported along microtubules in a KIF5B dependent manner. In contrast, KIF5B promotes tubulation from larger compartments (eg, autolysosomes). The authors proposed that this size‐dependent effect is driven by differences in surface tension triggered by the membrane's curvature.^112^

PtdIns(4,5)P_2_ plays a dual role in ALR. On one hand, it controls tubule formation as described above, on the other hand, it is required for the fission of newly formed proto‐lysosomes. This second function was uncovered with the observation that deficiency in PIP5K1A, a second kinase generating PtdIns(4,5)P_2,_ is characterized by the presence of long, stable reformation tubules without vesicle budding.^110^

Surprisingly, recent work revealed that PtdIns(4,5)P_2_ negatively regulates autophagosome‐lysosome fusion. In brief, its production triggers the dissociation of Rab7 and PLEKHM1 from the organelle membrane.^113^ Whether PtdIns(4,5)P_2_ activates a Rab7 GAP remains to be formally proven. In any case, PtdIns(4,5)P_2_ is emerging as pivotal player in the control of both fusion and reformation events actively shifting the balance towards the latter.

Another critical aspect of ALR is the retention mechanism that prevents diffusion of cargo and/or regulatory proteins from the parental autolysosome to the forming proto‐lysosome. In PI4KIIIβ depleted cells, lysosomal luminal components were aberrantly found in reformation tubules indicating that PI4KIIIβ actively contributes to retention.^114^ In addition, PI4KIIIβ ablation leads to hypertubulation that could only be rescued by expression of the catalytically active form of PI4KIIIβ. These data imply that PI4KIIIβ's control of lysosome tubulation and cargo retention is mediated by PtdIns(4)P.^114^

Disturbances in ALR are currently linked to hereditary spastic paraplegia and Parkinson disease. In brief, mutations in spastizin and spatacsin trigger the two most common forms of autosomal recessive hereditary spastic paraplegia. Their ablation leads to the accumulation of enlarged autolysosomes at the expense of lysosomes, a phenotype typical of ALR.^115^ In addition, spatacsin knockout mice display loss of cortical neurons and Purkinje cells consistent with a spastic paraplegia‐like phenotype.^116^ Although not formally proven, it has been proposed that ALR defects may result in a reduction of functionally active lysosomes that in turn would reduce autophagic clearance. The subsequent accumulation of undigested cargo would culminate in neuronal death. In a similar manner, Magalhaes et al proposed that in glucocerebrosidase (Gcase) deficient cells impaired ALR leads to a depletion of functional lysosomes capable of sustaining autophagic clearance of α‐synuclein.^117^ Of note, mutations in the glucocerebrosidase gene are causative for Gaucher disease (GD) the most common lysosomal storage disorders (LSD), and increase the risk of developing Parkinson disease.^118^, ^119^, ^120^

## 5 RAGS, LAMTOR, BORC AND ORGANELLE SIZE

Lysosomal mTORC1 activation is triggered as a coordinated response to nutrients/amino acids availability (through the Rag GTPases signaling pathway^121^, ^122^, ^123^), growth factors (through inhibition of the Tuberous Sclerosis complex [TSC] and activation of Rheb^124^, ^125^), and the cellular energy status (through AMP‐activated protein kinase [AMPK] phosphorylation of TSC2 and Raptor^126^, ^127^).

Interestingly, the Rag GTPases' physiological importance may go beyond recruiting and activating mTORC1 on lysosomes. Indeed, in cardiomyocytes, Rags play a so far underappreciated role in the regulation of lysosomal size. Knockouts of Rags as well as of a regulatory protein called GPR137B, cause an increase in numbers and size of lysosomes.^128^, ^129^ Kun‐Liang Guan et al nicely demonstrated that in RagA and RagB depleted cardiomyocytes, these changes are accompanied by a defect in acidification triggered by decreased lysosomal V‐ATPase levels.^128^ Of note, mTORC1 activity is not significantly compromised in these cells, although they display a constitutive activation of TFEB with persistent upregulation of the entire coordinated lysosomal expression and regulation (CLEAR) network. Together, these data indicate that the effect on the V‐ATPase is very specific. It remains to be seen if the regulation of lysosomal size observed in these cells is a general phenotype or represents a cell type specific phenomenon. Either way, it is remarkable that the phenotype of RagA and RagB knockout cardiomyocytes strongly resembles that of LSD,^128^ as discussed below. Furthermore, knockout of GATOR2 components also impairs lysosomal acidification and triggers an increase in the number of late endocytic organelles.^130^

The late endosomal/lysosomal adaptor and MAPK and MTOR activator (LAMTOR), also known as Ragulator, is a pentameric complex that recruits the Rags to the lysosomal membrane, a pre‐requisite for mTORC1 activation.^131^ Interestingly, LAMTOR knockout cells display increased numbers of late endosomes, lysosomes and autolysosomes that are significantly smaller than their counterparts in control cells.^26^, ^132^, ^133^ Furthermore, the acidification of lysosomes is apparently normal in LAMTOR depleted cells^133^, ^134^ and they display an increase in late endosomal proteins characteristic of the nuclear translocation of TFEB, constitutive activation of the CLEAR network and a concomitant rise in basal autophagy.^133^ Taken together, based on the currently available reports addressing organelle size, LAMTOR and the Rag GTPases epistatically contribute to mTORC1 activation, but their effects on lysosomal size are apparently (diametrically) opposing.

The BLOC‐1 related complex (BORC) contains eight subunits (Myrlysin/LOH12CR1, Lyspersin/C17orf59, Diaskedin/C10orf32, KxDL1, MEF2BNB, BLOS1, BLOS2 and Snapin) and was initially found to regulate lysosomal positioning.^135^, ^136^ In 2017, others and we have shown that LAMTOR associates with BORC, negatively regulating BORC and Arl8b dependent transport of lysosomes to the cell periphery.^137^, ^138^ Interestingly, BORC knockout cells display a multilayered deregulation of lysosomal homeostasis. In brief, the most conspicuous defect of BORC ablation is the accumulation of lysosomes in the perinuclear region.^135^ Later, it was also found that BORC knockouts show increased autophagic structures and a defect in autophagosome‐lysosome fusion^139^ (Figure 2D). In brief, depletion of BORC impaired the recruitment of the HOPS tethering complex to lysosomes (mediated by Arl8b^140^) and the subsequent assembly of the STX17‐VAMP8‐SNAP29 *trans*‐SNARE complex involved in autolysosome formation. In addition, the positioning defect observed in these cells implied that lysosomes were no longer mobile and therefore unable to translocate to the cell periphery where most autophagosomes originate. As such, the mobility defect translated into compromised organelle fusion.^139^ Recently, Snouwaert et al described a mouse mutant of the BORC subunit Diaskedin with dystrophic axonopathy and motor impairment. The Diaskedin Q87X mice showed swollen axons containing masses of unidentified membrane remnants.^141^

In addition to the already described phenotypes, we have recently uncovered that deletion of the BORC subunits Myrlysin and Diaskedin, compromises assembly of the complex on the organelle membrane and triggers a decrease in lysosomal size.^30^ BORC regulates PIKfyve‐dependent production of PtdIns(3,5)P_2_ actively controlling lysosomal reformation. This process requires AMPK, a known PIKfyve activator,^30^ and is additionally dependent on LAMTOR/Ragulator complex (Figure 2B). Interestingly, both LAMTOR and BORC knockout cells showed reduced lysosomal size, reaching only about 75% of those in WT cells.^26^, ^30^ Of note, the reduction of lysosomal diameter was associated with increased lysosomal frequency^26^, ^30^ probably reflecting some kind of physiological compensation.

Finally, it is worth mentioning that we observed a further, additive effect on the size reduction of lysosomes in LAMTOR plus BORC double knockouts.^30^ These data imply the existence of two independent mechanisms contributing to the observed size regulation.

## 6 DISEASE CONDITIONS WITH ALTERED ORGANELLE SIZE

Below, we describe disease conditions with reported alterations of endolysosomes, autolysosomes or lysosomes. Although we tried to follow in this article the above‐mentioned nomenclature, for some of the referred studies, it is unclear to which of the three classes of organelles the authors precisely refer to. Therefore, we decided to keep the organelles' terms as close as possible to the original descriptions by the respective authors. In addition, for a number of the reported diseases, it remains unclear how alterations of the organelles' sizes are mechanistically linked to the respective phenotypes. Nevertheless, by reporting and summarizing such cases, we would like to encourage colleagues and readers to address these open questions.

### 6.1 Lysosomal storage disorders

LSD is the name given to a group of approximately 50 genetic diseases caused by deficiencies in lysosomal and non‐lysosomal resident proteins; such deficiencies trigger the accumulation of disease specific substrates on lysosomes and other cellular locations. Thus, the most characteristic histological feature of LSD is the presence of enlarged lysosomes filled with undigested material.^142^, ^143^, ^144^ Depending on the enzymatic activity impaired in each disease type, LSD have been subdivided into mucopolysaccharidoses, sphingolipidoses and glycoproteinoses.

Nowadays, it is clear that the spectrum of LSD must also include other genetic alterations that disturb the synthesis and/or transport of lysosomal proteins and cargo and that are causal for the characteristically enlarged structures. Independently of its origin, the accumulation of nondegradable material within lysosomes has a profound impact on the organelle's physiology, size, trafficking and overall degradative capacity.^145^, ^146^, ^147^, ^148^ As an example, sphingomyelin accumulation in Niemann‐Pick disease cells, blocks TRPML1 and calcium‐dependent lysosomal functions.^149^

In general, defects on soluble, luminal proteins in lysosomes are more frequent than those triggered by depletion of lysosomal membrane proteins.^150^ The lysosomal‐associated membrane protein 2 (LAMP2) is a transmembrane protein specifically associated with endolysosomal organelles. Mutations in its gene are causative for an X‐linked dominant condition known as Danon disease.^151^ Patients present cardiomyopathy (hypertrophic or dilated), myopathy and mental retardation. The pathological hallmarks of Danon disease include the accumulation of autophagosomes and glycogen in cardiac and skeletal muscle cells.^151^ As such, Danon disease can be classified as an LSD caused by a non‐enzymatic lysosomal protein with a critical role in chaperone‐mediated autophagy.^152^, ^153^

Although the characteristics of the different diseases are very diverse, four pathophysiological hallmarks are found in common among several LSD: (a) inflammation, (b) altered calcium homeostasis, (c) lysolipid accumulation and (d) impaired autophagosome maturation.^150^

The galactosylceramidase enzyme is directly responsible for the degradation of galactosylceramide and psychosine. A subset of LSD, called lipidoses, display aberrant storage of lactosylceramide derivatives in the endosomal system. Their uptake and sorting to the Golgi apparatus is dependent on intracellular cholesterol levels.^154^ This and other observations led to the early recognition that altered lipid synthesis and trafficking contribute to LSD pathology and the characteristic lysolipid accumulations.^155^, ^156^, ^157^ Interestingly, a reciprocal regulation has also been observed in oligodendrocytes and astrocytes from Krabbe disease patients in which galactosylceramidase deficiency leads to psychosine accumulation that in turn triggers a significantly down‐regulation of AMPK activity. This cascade of events culminates in increased biosynthesis of lipids including cholesterol and free fatty acids.^158^

Another hallmark of LSD is the accumulation of calcium in the cytosol and depletion of ions from the endoplasmic reticulum (ER). Although aberrant calcium homeostasis is indeed a common denominator in this disease group, the underlying pathophysiological mechanisms differ among various LSD and can, at least in part, be discriminated based on the accumulated lipids. Whereas glucosylceramide sensitizes the ryanodine receptor to mediate calcium release from the ER,^159^ GM1 and GM2 ganglioside accumulation inhibits the reuptake of calcium to the ER by interfering with the function of the responsible transporter (SERCA).^160^ In addition, the increase in sphingomyelin observed in Niemann‐Pick type A disease triggers a severe reduction of SERCA expression levels, with concomitant accumulation of calcium in the cytoplasm.^161^ Finally, mucolipidosis type IV (MLIV) is a rare LSD triggered by loss of function mutations in mucolipin 1 (*TRPML1*), a lysosomal membrane channel releasing cations.^162^, ^163^ First described by Berman et al,^164^ MLIV displays a profound degenerative profile with neurodevelopmental‐, psychomotor‐, ocular‐ and gastric‐abnormalities.^165^, ^166^

### 6.2 Francois‐Neetens Mouchetee fleck corneal dystrophy (CFD)

CFD is an autosomal dominant disease characterized by the presence of white flecks distributed throughout all layers of the corneal stroma that do not affect vision. Mutations in PIKfyve have been implicated as the underlying cause of the disease.^167^ Although the relatively mild symptoms of CFD might at first glance appear inconsistent with the broad spectrum of PIKfyve functions, one should not forget that patients are heterozygous, carrying also a normal allele. Importantly, it has been known for a few decades that the corneal flecks characteristic of CFD correspond to swollen, vacuolated corneal fibroblasts (keratocytes) filled with mucopolysaccharides and complex lipids.^168^ This histological characterization is largely reminiscent of the phenotypes observed under compromised PIKfyve function.

### 6.3 Charcot‐Marie‐Tooth

Charcot‐Marie‐Tooth disease is a genetically heterogeneous motor and sensory peripheral neuropathy caused by mutations in more than 30 genes. In 2007, Meisler et al identified an autosomal recessive form of the disease, designated CMT4J that is caused by mutations in sac3 *(Fig4)*, the PtdIns(3,5)P_2_ phosphatase that takes part in the PAS complex.^74^ In the same publication, the authors also describe a “pale tremor” phenotype in mice generated on a mixed inbred strain background. These mice, genotypically characterized as sac3 *(Fig4)* KO, display a multi‐organ disorder with peripheral neuronopathy, central nervous system degeneration, and diluted pigmentation. Histological analysis revealed the accumulation of large vacuolar compartments, immunoreactive to LAMP2.^74^

Interestingly, analysis of human fibroblasts derived from CMT4J patients led to the observation that the intracellular movement of organelles is regionally impaired by the presence of large endolysosomal structures.^169^ No trafficking abnormalities were seen in the cell periphery. These data provide further evidence for a link between organelle size and motility.

The physiological importance of PtdIns(3,5)P_2_ was further corroborated in neurons and astrocytes of mice with mutations in either sac3 (Fig4) or arPIKfyve *(Vac14)*. In brief, expression of Fig4 in neurons is necessary and sufficient to prevent spongiform degeneration, whereas expression in astrocytes prevents the accumulation of autophagy markers and microgliosis seen in these cells, but has no impact in the neuronal phenotype.^170^, ^171^ Taken together, these results imply that autophagic abnormalities play a secondary role in the etiology of CMT4J and that disease therapy should target neurons directly. Interestingly, sac3 *(Fig4)* nonsynonymous mutations were also described in approximately 2% of patients with amyotrophic lateral sclerosis (ALS) an incurable motor neuron disease, and in primary lateral sclerosis (PLS) as well.^172^ In 2014, Colin Martyn and Jun Li provided a comprehensive review on Fig4 deficiency phenotypes and subjacent mechanisms that culminated with the proposal that the deficiency of *Fig4* in humans and mice likely corresponds to a new form of LSD.^173^

### 6.4 Frontotemporal lobar degeneration with TDP‐43 inclusions

Frontotemporal lobar degeneration with TDP‐43 inclusions (FTLD‐TDP) is a fatal neurodegenerative disease distinguished by the accumulation of inclusions of hyperphosphorylated and ubiquitinated TAR DNA‐binding protein of 43 kD (TDP‐43), in glia and neurons. The disease profile is accompanied by progressive loss of neurons. Variants of the lysosomal protein TMEM106B are associated with FTLD‐TDP risk.^174^ TMEM106B is expressed in several cell types including neurons and microglia,^175^ and it is known that in cortical neurons TMEM106B expression correlates with neuronal maturation.^176^ In neurons, TMEM106B knockdown reduces the number of lysosomes. In a reverse manner, TMEM106B overexpression inhibits organelle transport and leads to the accumulation of large LAMP positive lysosomes in the soma.^175^ These data led Stagi et al to propose TMEM106B as a key determinant of lysosomal size in neurons,^175^ highlighting the importance of lysosome homeostasis in neurodegenerative disorders. Two different pieces of evidence give a hint at the probable mode of action: First, inhibition of the V‐ATPase increases TMEM106B expression levels, linking acidification to the regulation of TMEM106B.^177^ Second, the levels of TMEM106B strongly regulate the nuclear translocation of TFEB.^175^ This tight connection led Stagi et al to additionally propose that TMEM106B control of TFEB nuclear translocation and concomitant activation of the CLEAR network might provide the mechanistic explanation for the observed changes in lysosomal size and number.^175^

### 6.5 Chediak‐Higashi syndrome

In 1943, Beguez‐Cesar reported the premature death of three siblings from a consanguinity marriage. The children displayed hepatosplenomegaly, granular leucocytes, partial albinism and suffered from recurrent infections. This case is taken as the first description of Chediak‐Higashi syndrome (CHS). It is a rare, autosomal recessive disorder associated with progressive neurologic dysfunction, increased susceptibility to infections (skin, respiratory tract and mucous membranes) and oculocutaneous albinism (OCA). The morbidity associated to CHS is often a consequence of recurrent infections and in most cases is preceded by an accelerated phase of the disease characterized by a lymphoproliferative syndrome with hemophagocytosis, infiltrating major organs.^178^

The presence of inclusion bodies in several immunological cell types, including macrophages, T‐lymphocytes, neutrophils and leucocytes serves as diagnostic marker for CHS.^179^ Furthermore, it is clear that a defective structure or function of melanosomes in melanocytes is causing albinism, 180 and that the mild bleeding tendency observed in these patients can be explained by the absence of platelet dense bodies.^180^, ^181^ Taken together, CHS pathophysiology roots on defects in the maturation of lysosomes, phagosomes and lysosomal‐related organelles (LRO; eg, melanosomes, cytolytic granules and dense granules), leading to the accumulation of enlarged lysosomes and LRO that cannot be secreted.^182^, ^183^

CHS is caused by mutations in the *LYST* (lysosomal trafficking regulator) gene. The enormous size of the LYST protein has been a significant hurdle to those attempting to shed light on its function. Nevertheless, data from LYST homologs in *Drosophila melanogaster* and *Dictyostelium discoideum* indicate that LYST might regulate organelle fusion.^184^, ^185^, ^186^ Unfortunately, the neurologic phenotypes displayed by LYST‐mutant mice were found to depend on genetic background.^187^ Nevertheless, the characterization of *Lyst*‐mutant *beige* mice indicated that LYST controls phagosomal maturation, triggered as a response to bacterial infection. It specifically regulates the formation of endolysosomal/phagosomal compartments required for the activation of TIR domain‐containing adapter‐inducing interferon β (TRIF) dependent Toll like receptor signaling.^182^ These findings provide a first mechanistic explanation for the impaired innate immune response observed in CHS patients. A pilot publication used small interfering RNAs to address LYST function in human cells. In brief, the authors confirmed the enlarged lysosomes characteristic of CHS but did not observe compromised autophagy or defects in endocytic transport and degradation.^188^

### 6.6 Treatment options for LSD and related diseases

There is light at the end of the tunnel for those suffering from LSD. We have seen a rapid increase in our understanding of the processes regulating lysosomal homeostasis and function, as well as on the molecular basis of LSD and their associated pathophysiology. In parallel, orphan drug legislations that have been passed in the United States and Europe over the last two decades, encouraged Biotech companies to invest on the treatment of rare conditions. We are now in a position where treatment options are no longer restricted to hematopoietic stem cell transplantation or enzyme replacement therapy but may nowadays include substrate reduction drugs or small molecule pharmacological chaperones. Furthermore, clinical trials are currently under way for the use of gene therapy approaches both in vivo and ex vivo and for the use of drugs regulating stop codon read through. For reviews on the treatment of LSD, we refer the readers to References 189, 190, 191, 192.

Over the last decade, several groups have shown that TFEB overexpression improves lysosomal trafficking, autophagosomal‐lysosomal fusion and exocytosis, in a number of LSD and neurodegenerative storage disorder models.^193^, ^194^, ^195^, ^196^ These “proof of concept” experiments route on promoting nuclear translocation of members of the MiT/TFE family of transcription factors to increase autophagy, which might alleviate the lysosomal accumulation of undegraded materials characteristic of the diseases. However, a word of caution has to be raised here because it is also known that TFEB and other members of the MiT‐TFE family display oncogenic features.^197^, ^198^, ^199^ Nevertheless, recent reports on trehalose, a natural sugar known to induce TFEB expression, indicate that treatment reduces disease burden in a number of pre‐clinical LSD models.^196^, ^200^ Importantly, trehalose treatment significantly reduced the number of large autophagic vacuoles in the cortex and cerebellum (neurons and microglia) of MPS IIIB mice.^200^ Since the accumulation of large autophagic vacuoles is considered as the primary cause of the neuronal degeneration observed, these results serve as an example that therapies triggering organelle size reduction could be efficient tools in the treatment of LSD. Expanding on the TFEB concept, inhibitors targeting mTORC1 on lysosomes could be relevant treatment options in the future.^201^

Following the precedent created by small molecular weight compounds targeting the cystic fibrosis transmembrane conductance regulator (CFTR) channel, scientists at the University of Munich have targeted TRPML1, the endolysosomal cation channel mutated in Mucolipidosis type IV.^202^ The authors identified a small molecule that restores the phenotype of a specific subset of TRPML1 mutations. This agonist, known as MK6‐83, restores TRPML1 activity, endolysosomal trafficking and heavy metal homeostasis. Strikingly, not only the number but also the size of Zinc accumulating lysosomes was markedly reduced upon MK6‐83 treatment.^202^ It remains to be seen if MK6‐83 or related molecules make it to an approved therapy. In any case, scientists are a step closer into bringing personalized medicine to the treatment of LSD. Despite this justified optimism, it remains a pressing goal to translate the accumulating knowledge on the pathophysiology of LSD into better treatment options. Those may in part arise from the complementary use of several of the recently developed therapeutic approaches.

## 7 CONCLUDING REMARKS: PERSPECTIVES

### 7.1 Does lysosomal size really matter?

Size, in our point of view, is a morphological readout of organelle homeostasis. As such, size is tightly maintained by the coordinated action of a large number of factors directly or indirectly regulating lysosomal fusion, fission and frequency. These include, but are not restricted to, the V‐ATPase, phosphoinositides, mTORC1 signaling, coating and tethering factors, SNAREs, calcium transporters, actin and small GTPases. This impressive list of regulators of lysosomal size is far from being complete. As an example, recent work on organelle contact sites underscored their involvement in the regulation of size: ER contact sites define the position and timing of fission of early and late endosomes, whereas mitochondria‐lysosome contacts were shown to promote Rab7 hydrolysis thereby regulating lysosomal size.^203^, ^204^

### 7.2 What is the contribution of lysosomal size to disease?

**Figure 3:**
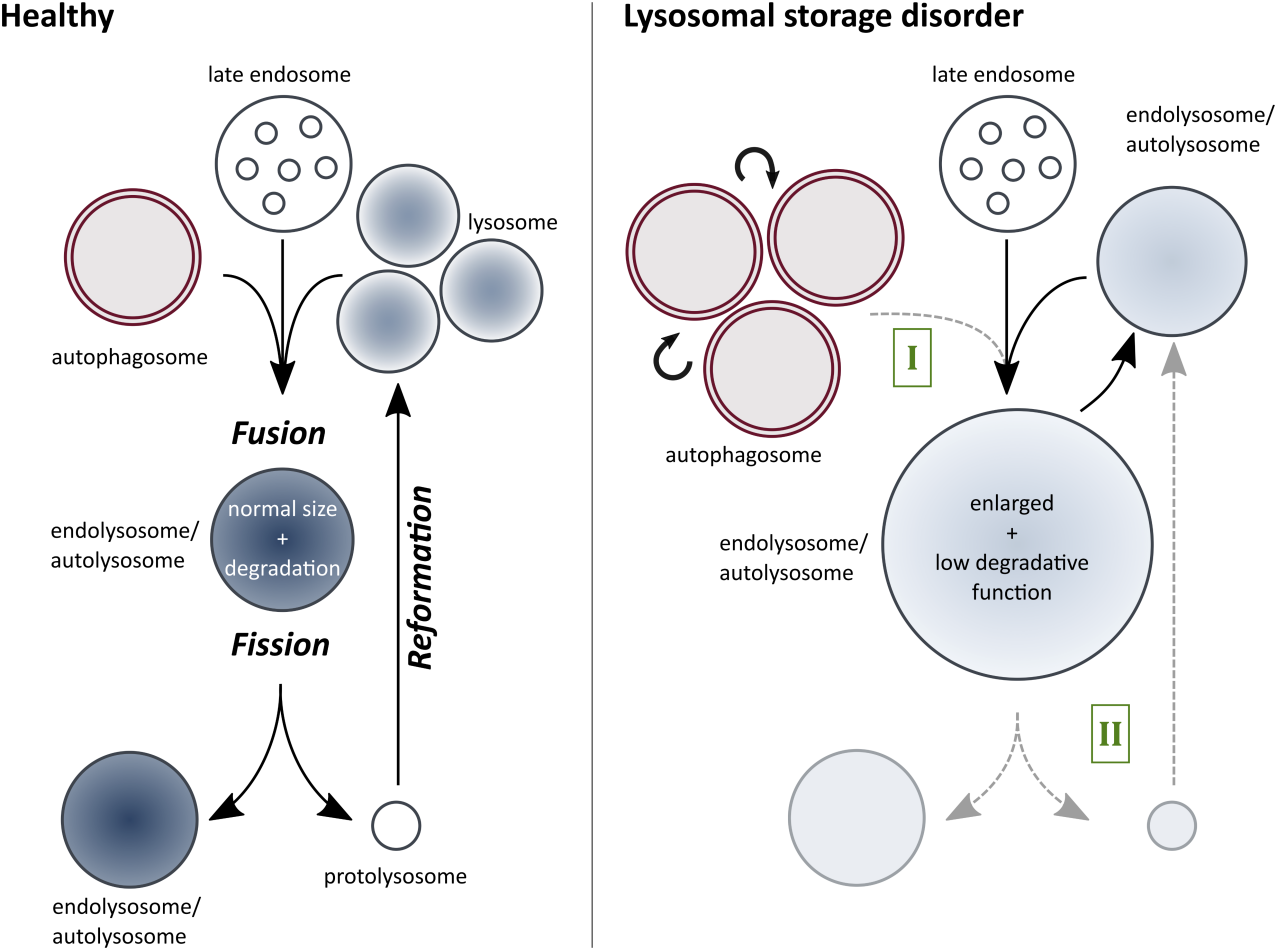
Model summarizing the working hypotheses underlying LSD pathophysiology. Under healthy conditions (left part of the scheme), correct acidification, fusion and fission events maintain the organelle's function and morphology. In contrast, LSD display alterations of the regulatory mechanisms controlling the organelle's size (right part of the scheme), either as an impairment of autophagosome‐lysosome fusion (highlighted in green, I) and/or as a reduction of lysosomal reformation (highlighted in green, II) that subsequently lead to the accumulation of autophagic compartments and enlarged endolysosomes. These alterations are accompanied by acidification (blue color gradient) defects that promote the accumulation of undegradable cargo in catabolically inactive organelles. Finally, these events are amplified in a feedforward loop that culminates in the progressive phenotype characteristic of LSD

A decade ago, it was proposed that LSD pathophysiology might root on reduced fusion efficiency between autophagosomes and lysosomes.^146^ This block in autophagy would activate a compensatory feedback mechanism increasing autophagosome formation (Figure 3 right side, annotated in green as I). The variation in the severity of the symptoms expressed in the different LSD would depend on the strength of the fusion block and on the extent of the expansion of autophagosome/lysosomal compartments generated as a consequence.^150^ A second hypothesis raised to explain LSD progression, is based on impaired lysosomal reformation (Figure 3 right side, annotated in green as II). In brief, fibroblasts from patients with different LSD (Scheie syndrome, Fabry disease and Aspartylglucosaminuria) show compromised mTOR reactivation and ALR defects.^22^ The tight interplay between lysosomal catabolism and ALR would explain why minor defects in either of two processes are amplified in a positive feedback cycle, eventually triggering the progressive pathology of LSD.^205^

These hypotheses have two aspects in common. First, independently of the initiating mechanism, the secondary progressive accumulation of nondegradable cargo (from damaged organelles to ubiquitinated proteins) seems to play a pivotal role in LSD pathology. Second, both of the mechanisms proposed control the organelle's size. In Figure 3 we present a model that tries to summarize the working hypotheses underlying LSD pathophysiology, including fusion, fission and acidification defects, cargo accumulation and increased organelle size.

The emerging mechanistic and phenotypical similarities between LSD and other diseases, in particular those with increased lysosomal size, highlight the need to reconsider disease boundaries.^191^ In all of these disorders, independently of the initiating factor, the intertwined dynamic balance between fission, fusion, and number of lysosomes is impaired and actively contributes to disease progression. To cut it short, lysosomal size does indeed matter.
